# Performance Assessment of Asphalt Mixture Produced with a Bio-Based Binder

**DOI:** 10.3390/ma14040918

**Published:** 2021-02-15

**Authors:** Elena Gaudenzi, Francesco Canestrari, Xiaohu Lu, Fabrizio Cardone

**Affiliations:** 1 Department of Civil and Building Engineering and Architecture, Università Politecnica delle Marche, Via Brecce Bianche, 60131 Ancona, Italy; f.canestrari@staff.univpm.it (F.C.); f.cardone@staff.univpm.it (F.C.); 2 Nynas AB, SE-149 82 Nynäshamn, Sweden; xiaohu.lu@nynas.com

**Keywords:** bio-binders, asphalt concrete, mechanical characterization

## Abstract

Nowadays, the growing energy costs and pressing worldwide demand for petroleum-based products create a strong need to develop alternative binders deriving from green and renewable sources. Bio-binders (or bitumen added to bio-based materials) can potentially be a viable alternative for the production of bituminous mixture, promoting the circular economy as well as environmental sustainability principles without reducing the overall performance of the mixture. In this context, the current study focuses on evaluation of the effects of a bio-binder on the mechanical response of asphalt concrete (AC) produced with it. In particular, a 10% bio-oil deriving from a by-product of the paper industry has been blended with a conventional 50/70 penetration grade bitumen to obtain the bio-binder. Moreover, plain bitumen having the same consistency was chosen to produce a reference AC. Two dense-graded AC wearing courses were prepared in the laboratory according to Italian technical specifications. A mechanical characterization in terms of indirect tensile strength, indirect tensile stiffness modulus, fatigue response and permanent deformation resistance was performed on gyratory compacted specimens using both conventional and performance tests. In addition, aging and water sensitivity of the AC specimens were evaluated. Overall results highlight that the AC produced with the bio-binder did not show reduced mechanical properties and it was comparable to the reference AC regardless of aging and water conditioning. This highly encourages the use of bio-binder as a viable alternative in asphalt technology.

## 1. Introduction

Performance of asphalt pavements depends on the quality of materials employed and the accuracy achieved during design and construction phases. Materials characterization is one of the most important tasks in the design of the pavements, from which both their mechanical properties and durability throughout their service life derive. Asphalt mixture is a complex composite that consists of solid aggregates, mineral filler, bitumen and air voids. Although the bitumen plays a very important role, the comprehensive performance of the asphalt mixture is determined by the set of the whole component phases. Since conventional binders are derived from the petroleum refining process, which is a non-renewable and highly impacting resource, one of the main current efforts is to move towards “greener” solutions, with the aim to embrace the principles of environmental sustainability and circular economy [1]. Contrarily to the linear economy, in fact, which is based on the “make–use–dispose” model of resources’ consumption, the circular economy meets the principles of minimizing of waste, emissions and energy leakage and repair, reuse, remanufacturing, refurbishing and recycling [2]. This recent development is gaining increasing interest from the asphalt pavement industry, which is moving towards the use of bio-binders. Bio-binders can be defined as bituminous binders in which bitumen is entirely or partially replaced with various bio-resources, and depending on the percentage of biological origin added to the conventional binder, it is possible to talk about direct alternative binders (100% replacement), bitumen extender (25% to 75% bitumen replacement), and bitumen modifier (<10% bitumen replacement) [3].

Various bio-resources, such as waste wood, animal waste [4], soybean straw [5] and waste cooking oil, have been identified as suitable for reducing the production of petroleum-based asphalt for the sustainable developments of flexible pavements [6]. The suitability of bio-resources is a key point for the production and employment of bio-binders, and can be assessed through the FTIR method, which can evaluate the compatibility of a substance or bitumen additive and its structural stability [7,8].

Some research works have been conducted on performance evaluation of bio-binders from a chemical, morphological and rheological point of view. Specifically, several studies with a focus on the healing potential, fatigue behavior, as well as aging properties, demonstrated promising results, highlighting no compromising behavior or particular drawbacks and, in some cases, improved response as well [9,10,11,12].

Recent studies also suggested the use of bio-oil as an additive, modifier or extender of bitumen, also combined with the use of polymers [13] or crumb rubber from used tires [14], to enhance the overall performances besides the reduction of pavement’s carbon footprint.

However, not much is yet known about the application of bio-binders in asphalt mixtures. Yang et al. investigated polymer-modified bitumen containing bio-oil and found appreciable results in terms of fatigue, rutting and thermal cracking resistance [15]. Further mechanical characterizations were conducted on bio-oil-modified asphalt mixtures aimed at investigating the performances at low, medium and high temperatures. In particular, Peralta et al. found that combining asphalt tire rubber and a vegetal bio-oil creates mixtures that can resist fracture cracking at low temperatures with their high stiffness [16]. Ma et al. reported that the relationship between flexural modulus and fatigue life was more suitable for mixtures with bio-binders [17,18] whereas Song et al. indicated that bio-asphalt improved the high-temperature performance of the mixture [19].

Therefore, on the basis of previous investigations of performance comparison between a bio-binder and a reference conventional bitumen having similar consistency [9,20], this study aims at evaluating the effects of such bio-binders on the behavior of an asphalt mixture. To this end, a mechanical characterization was carried out on the asphalt mixture in terms of indirect tensile strength, indirect tensile stiffness modulus, fatigue (i.e., resistance to cyclic loading), resistance to fracture and permanent deformation. In addition, long-term aging effects and water sensitivity were taken into account.

## 2. Materials and Specimen Preparation

In this study, two dense-graded asphalt concretes (AC) for wearing courses with nominal maximum aggregate size of 14 mm were produced according to the Italian national technical specifications for highways [21].

**Figure 1:**
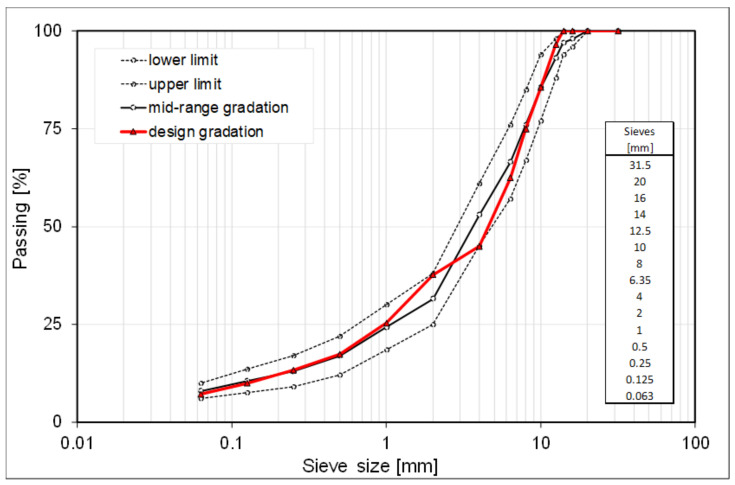
Aggregate gradation.

The aggregates employed were a mix of two fractions of basalt coarse aggregate, a limestone sand and a calcareous filler, all collected from a local asphalt plant in Italy and combined to obtain the design gradation as shown in Figure 1.

The two ACs were produced by using a bio-binder and a plain bitumen, respectively, both at the same binder content of 5.8% (by aggregate weight). The bio-binder was prepared by adding 10% (by weight) bio-oil to a conventional 50/70 penetration grade bitumen through a mixer, setting blending speed at 500 rpm and the temperature at 150 °C for about 10 min [22]. The percentage of bio-oil has been defined on the basis of various tests performed, including chemical, morphological and rheological as well as conventional and viscosity tests. In particular, the renewable bio-oil was a residue generated in the processing of a by-product from the wood pulp and paper industry, whose compatibility and structural stability in bitumen has been assessed in previous studies through the FTIR method [11]. Specifically, the bio-oil was a complex mixture of numerous chemicals and consisted of rosin acids, fatty acids and neutral compounds. Compared to normal bitumen, which typically consists of about 80% to 85% carbon, 10% hydrogen, and 0.5% oxygen, the bio-oil contained a similar level of carbon and slightly higher hydrogen, resulting in higher H/C ratios than those for bitumen (about 1.2). It also contained much higher oxygen content, mostly in the form of acids and esters. A higher level of acids may be a concern in terms of corrosivity.

It should also be mentioned that, for a bituminous binder containing such bio-oil, a different smell can be noticed; but according to a previous report [23], the smell was not annoying or irritating. It was also reported that, in the fumes generated from the bio-binders in the laboratory at high temperatures, no compounds were detected at any level that triggered concerns of increased health risk for asphalt workers.

Since the addition of bio-oil to conventional bitumen causes a consistency reduction [22], the binder chosen as reference material was a softer plain bitumen, having a similar consistency to the bio-binder. Table 1 summarizes the main properties of both binders [20]. The bio-based and reference AC were coded as MB50/70 + A10 and MB120, respectively.

Preliminary viscosity tests by means of a rotational viscometer were performed on both binders to determine the production temperatures, ensuring an equi-viscosity condition during the preparation of the AC. According to the NCHRP 648 specification [24], viscosities of 0.17–0.19 and 0.31–0.33 Pa·s were adopted as reference value for the selection of the mixing and compaction temperatures of mixtures, respectively. Temperatures of 150 °C and 135 °C as mixing and compaction temperatures, respectively, satisfying viscosity requirements for both binders, were selected for both mixtures.

After mixing by an automated mechanical mixer, the loose asphalt mixture was aged in a forced-draft oven for 4 h at 135 °C to simulate the short-term aging of material. Subsequently, the short-term aged mixture was compacted by using a gyratory compactor (GC) according to the European standard EN 12697-31 [25] to obtain 100 and 150 mm diameter cylindrical specimens with a thickness of 60 and 150 mm, respectively. These heights were selected in order to have specimens characterized by a residual air voids content of 4 ± 0.5% (EN 12697-5).

According to the experimental program, two series of AC specimens were tested to evaluate the aging effect and water sensitivity. Long-term aging was simulated by placing a series of compacted specimens for five days at 85 °C in a forced-draft oven (AASHTO R30-02). In order to assess the water sensitivity a further series of compacted specimens was conditioned in a water bath at a temperature of 40 °C for 72 h (EN 12697-12).

## 3. Experimental Program

Experimental investigations were carried out on specimens tested at dry, wet and long-term aged conditions. Specifically, the mechanical characterization consisted of indirect tensile strength (*ITS*), indirect tensile stiffness modulus (*ITSM*), indirect tensile fatigue test (ITFT), semi-circular bending (SCB) and triaxial cyclic compression (TCC) tests. The testing campaign involved a total of 114 specimens and Table 2 sums up the experimental program in terms of number of replicates for each mechanical test.

### 3.1. Test Methods

#### 3.1.1. Indirect Tensile Strength

*ITS* tests were performed at 25 °C according to the EN 12697-23 standard. Specifically, a compression force was applied in a continuous manner along the two vertical diameters until the specimen reached failure. The *ITS* was calculated by the following equation:

(1) $$ITS=\frac{2P}{\pi Dh}$$

where *P* is the peak load applied, *D* and *h* are the specimen diameter and height respectively.

The tests were performed on the 100 mm diameter cylindrical specimens. Before testing, specimens were conditioned in a climatic chamber for at least 4 h at 25 °C. Four replicates for each testing condition were tested.

#### 3.1.2. Indirect Tensile Stiffness Modulus

*ITSM* tests were carried out by means of a servo-pneumatic machine according to EN 12697-26. The standard defines an impulsive load, with rise-time of 124 ms, to be applied to achieve the target horizontal deformation of 5 ± 2 μm. The *ITSM* was calculated by the following equation:

(2) $$E=\frac{F\left(\nu +0.27\right)}{z\cdot h}$$

where *F* is the compressive load applied along the vertical diameter of a cylindrical specimen, *z* is the maximum horizontal deformation, *h* is the specimen height and *ν* is the Poisson’s ratio at test temperature.

The tests were performed on 100 mm diameter cylindrical specimens, which were conditioned in a climatic chamber for at least 4 h before testing. For each AC and testing condition, 9 specimens were tested at 20 °C, whereas among these a series of 4 specimens were tested at a further two temperatures, 10 and 40 °C, with the aim of evaluating the thermal sensitivity of the investigated AC.

#### 3.1.3. Indirect Tensile Fatigue Test

After the *ITSM* test, 6 specimens were subjected to the ITF test in order to estimate the resistance to cyclic loading of AC. The tests were carried out at 20 °C in controlled strain mode according to EN 12697-24 and three different deformation levels (250, 300, 350 μstrain) were selected. The failure was established as the complete fracture of the specimen. Tests were performed on the specimens previously subjected to the modulus test (6 out of 9). The system measures the cumulative deformation of the specimen throughout the test and the number of loading cycles to failure.

Each specimen was conditioned in a climatic chamber at 20 °C for 4 h before testing, and two replicates for each deformation level for a total of 6 repetitions for each AC and testing condition were tested in order to depict the fatigue curve as an indicator of fatigue response of the material.

#### 3.1.4. Semi-Circular Bending Test

SCB tests were run to assess the cracking potential of the investigated AC. SCB tests were performed according to EN 12697-44 and consisted of applying a three-point bending load to a half-cylindrical specimen having a central artificial notch. The tests were conducted at a constant vertical deformation rate of 5 mm/min and the fractures started to propagate from the tip of the artificial notch through the “ligament area” where the tensile load was applied. The tests were performed at 10 °C and for each AC and testing condition, four replicate specimens were tested.

Shaped specimens for the SCB test, consisting of 4 half-cylindrical specimens of thickness of 50 mm, were obtained from a single 150 mm diameter cylindrical compacted specimen. An artificial notch (with 15 mm depth) was then cut in the middle of the base of each specimen (EN 12697-44). Before the test, specimens were kept in a climatic chamber at a temperature of 10 °C for at least 4 h.

The fracture toughness was calculated by the following equation:

(3) $$k_{Ic,i}=\sigma_{max,i}\cdot f\left(\frac{a_{i}}{W_{i}}\right)\;[N/mm^{3/2}]$$

where *W_i_* is the height of the specimen (mm), *a_i_* is the notch depth of specimen (mm) and *σ_max_* the stress at failure of specimen (N/mm^2^).

In addition, results were also analyzed in terms of fracture energy (*G*), determined as the whole area under the load-displacement curve normalized with respect to the area of ligament [26,27]. The parameter *G* (J/m^2^) represents the work required to increase the fractured surface until complete failure, and it was calculated as defined in Equation (4):

(4) $$G=\frac{\int Fds}{t_{i}\cdot \left(W_{i}-a_{i}\right)}$$

where ∫Fds is the area under the whole load-displacement curve and *t_i_* is the thickness of the specimen (mm).

#### 3.1.5. Triaxial Cyclic Compression Test

TCC tests were run to investigate the resistance to permanent deformations of the selected mixtures. TCC tests were carried out according to EN 12697-25 (test method B) at 60 °C. Specifically, 100 mm diameter and 60 mm-thick cylindrical specimens were placed between two plan parallel loading platens in a triaxial chamber and subjected to a confining pressure σ_c_ (set equal to 150 kPa) and a superposed cyclic axial block-pulse stress σ_a_(t) (set equal to 300 kPa) for 10,000 loading cycles. Before testing, specimens were subjected to a preconditioning time of at least 12 h at the test temperature. During the test, the cumulative axial strain of the test specimens was measured as a function of the number of load cycles and depicted to obtain the creep curve. Finally, the average creep rate (*f_c_*) was selected to evaluate the permanent deformation behavior of the mixtures (Equation (5)):

(5) $$f_{c}=\frac{\varepsilon_{n1}-\varepsilon_{n2}}{n_{1}-n_{2}}$$

where *ε_n_*_1_ and *ε_n_*_2_ are the cumulative axial strain of the test specimen after *n*_1_ and *n*_2_ loading cycles in percent (%), and *n*_1_ and *n*_2_ are the number of repetitive loading cycles, while the parameter *f_c_* is the slope of the (quasi) linear part of the least square linear fit and represents the rate of the permanent deformation increase.

## 4. Results and Discussion

### 4.1. Compactability

As abovementioned, all specimens were compacted by the GC imposing the final height in order to obtain specimen series characterized by a similar residual air voids content. Based on this approach, the number of rotations needed to end the compaction of each specimen was considered as an indicator of the mixture’s workability.

**Figure 2:**
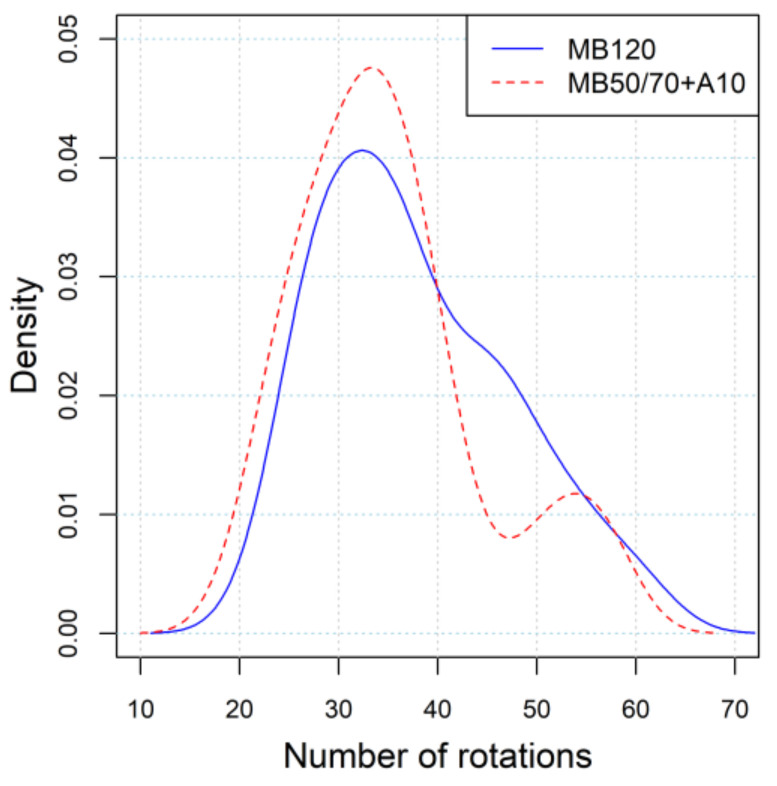
Empirical probability density function of number of rotations for the investigated mixtures.

A total of 96 specimens (48 for each AC) have been taken into account. Figure 2 shows the comparison of the empirical probability density functions of the number of rotations, N_GC_, for the two investigated mixtures. Moreover, the results of compaction in terms of average value and standard deviation of N_GC_ for both materials are summarized in Table 3 along with the outcome of a one-way ANOVA at 95% confidence level aimed at verifying the statistical significance of the results.

It can be observed that the density curves show a similar and almost overlapped trend, meaning that both specimen series basically required the same compaction energy. In particular, both distributions were characterized by a similar mean value and standard deviation, equal to 35.1 and 9.4 for the bio-mixture and 37.8 and 9.6 for the reference mixture, respectively. Moreover, results of the statistical ANOVA confirm that no significant difference existed in terms of compaction effort between the bio- and reference mixture and allows stating that the two asphalt mixtures were basically characterized by the same workability. Therefore, no direct effects due to the use of bio-binder can be seen on the compaction of the mixture, highlighting rather that compaction was strictly dependent on the consistency of the binder.

### 4.2. Indirect Tensile Strength

**Figure 3:**
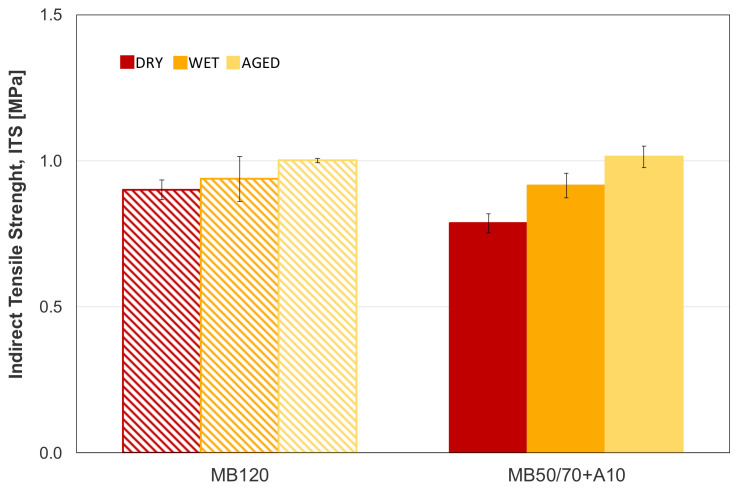
Indirect tensile strength results in the three testing conditions at 25 °C.

Figure 3 displays the *ITS* test results for the reference and bio-binder ACs. As far as dry conditions were concerned, the AC produced with bio-binder showed lower *ITS* as compared to the reference AC. It is believed that this result is not imputable to truly lower strength properties of MB50/70 + A10 but to the steric hardening effect. Indeed, because of laboratory constraints, fewer days elapsed between the compaction and test of MB50/70 + A10 specimens compared to a longer time spent for the B120 mixture. This finding is supported by the results gathered in the other testing conditions, where no significant difference in strength response can be noted between the two mixtures. In particular, it seems that water conditioning did not affect negatively the strength of either AC, on the contrary, a slightly improved *ITS* was observed with respect to dry conditions. This phenomenon can be explained considering that the investigated mixtures contained a very low level of air voids (4 ± 0.5%), not allowing water effects against binder-aggregate adhesion and, at the same time the wet conditioning acted as a sort of accelerated “curing” to which specimens were subjected. This result confirms previous studies, which claimed that the adhesive bond was not reduced but, on the contrary, it seems to even have improved adhesion in presence of water [11,28]. Aging conditions showed the highest *ITS* values, confirming the hardening effect experienced by materials during aging. However, no relevant differences between the two aged mixtures can be highlighted.

### 4.3. Indirect Tensile Stiffness Modulus

Table 4 summarizes *ITSM* results, obtained at three temperatures for each testing condition (i.e., dry, wet and aged). In particular, results are shown in terms of change in *ITSM* expressed as the ratio between wet and aged condition with respect to the reference dry one.

From the analysis shown in Table 4, it is possible to highlight that the ratio was always higher than 1. These results mean that in general water conditioning did not affect mechanical response, but rather, it resulted in a further conditioning leading to a stiffening of the material. Aging conditions show the highest values of the ratio, and confirm the effects of the aging process, which caused a global hardening of the AC. Overall, the ACs’ responses in terms of stiffness properties were quite similar because *ITSM* values of both investigated ACs can be considered comparable at all temperatures. However, it is right to note that in each testing condition MB50/70 + A10 showed higher values as compared to MB120, indicating a higher susceptibility of bio-based AC to hardening due to aging [29,30]. This effect is in contrast to what was observed in previous studies on the binder phase [10]; it could be due to the interaction between aggregates and binder, and could be an interesting point of reflection for future investigations.

**Figure 4:**
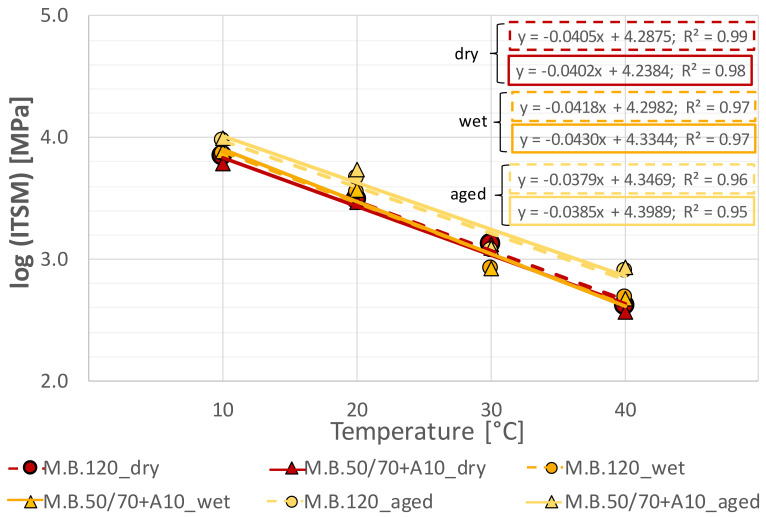
Comparison in terms of thermal susceptibility of asphalt mixtures.

For both ACs and each testing condition, Figure 4 depicts *ITSM* data fitted by a linear regression describing the change in *ITSM* as a function of temperature. In particular, the slope of the regression curve on the plane can be used to assess the temperature sensitivity of studied ACs: the higher the slope, the more temperature sensitive the mixture.

It can be observed that for both asphalt mixtures, regression curves in dry and wet conditions are overlapped and this result confirms that water conditioning had no significant effects regardless of the temperature; however, note that moisture sensitivity observed depended on the volumetric characteristics of specimens. Moreover, the investigated ACs showed a comparable temperature sensitivity in both dry and wet conditions.

As regards aging condition, the aged asphalt mixtures showed a significant increase in stiffness associated with a decreased slope; however, they were still very similar for the two ACs. Based on this finding, the lower slope under aged conditions means the materials experienced less temperature susceptibility, compared to in dry and wet conditions.

Overall, for the same test condition (dry, wet, aged) it can be highlighted that the bio-based AC showed temperature sensitivity comparable to the reference one without showing any particular effect related to the type of conditioning.

### 4.4. Indirect Tensile Fatigue Test

**Figure 5:**
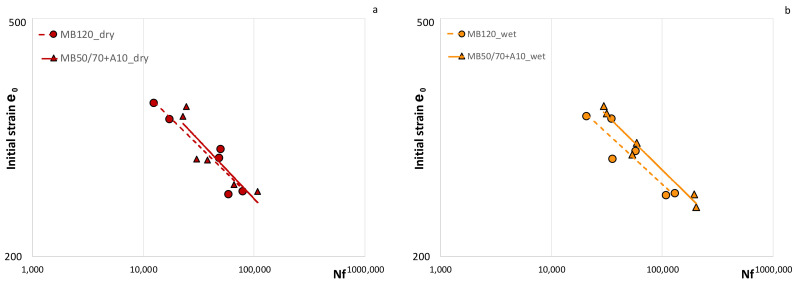

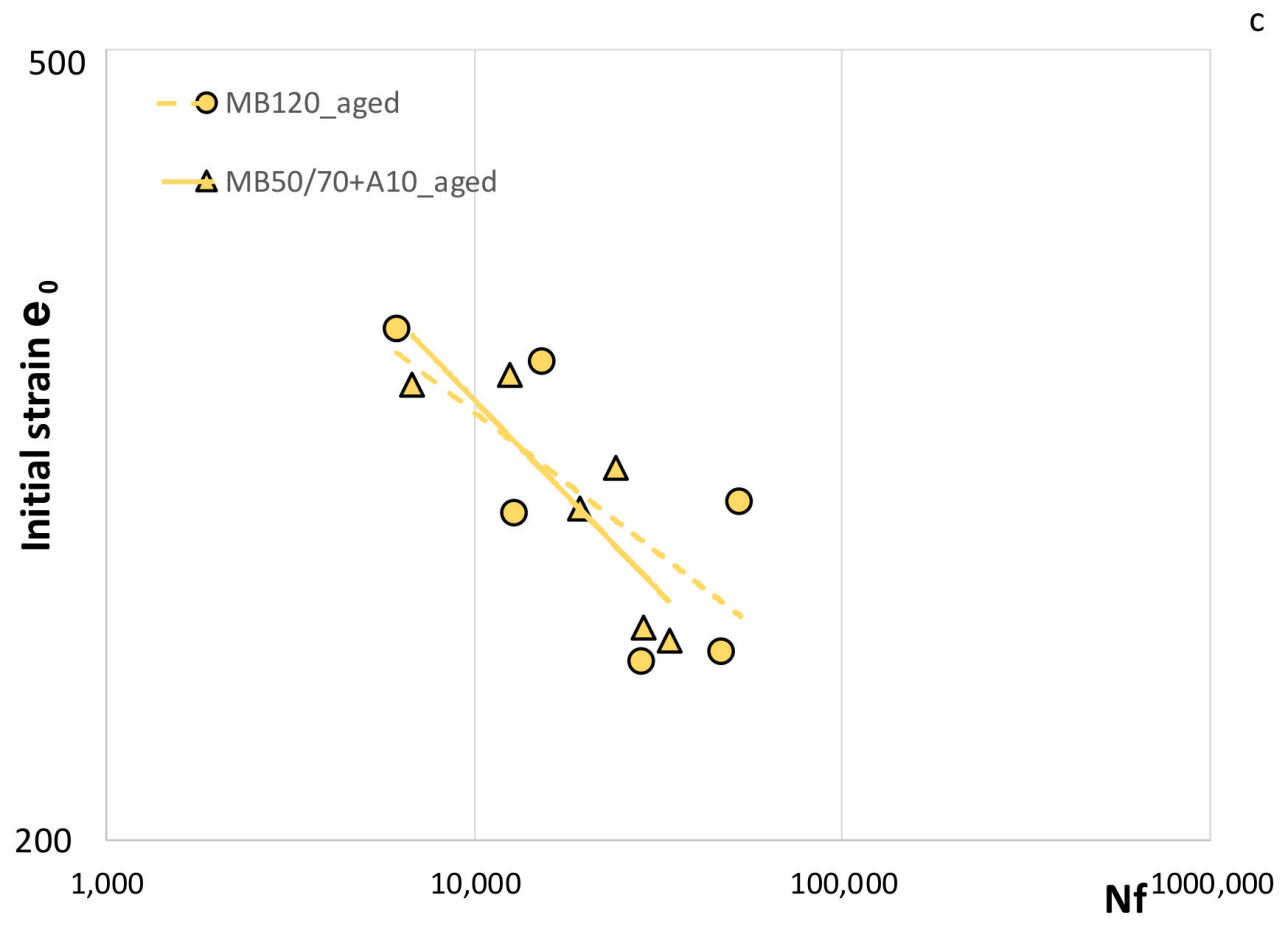
Fatigue laws of materials: (**a**) dry conditions, (**b**) wet conditions, (**c**) aged condition.

Figure 5 displays the fatigue behavior of the bio-based AC compared with the reference mixture in dry, wet and aged conditions and Table 5 sums up the equations of the fatigue laws. It is worth noting that both mixtures, having the same gradation and binder content, showed very similar fatigue response under the same test conditions.

In particular, the behavior of the bio-based AC in dry conditions can be considered clearly comparable to that of the reference one, while in wet conditions MB50/70 + A10 seemed to show a slightly improved fatigue behavior compared to the reference MB120 mixture. In aged condition, both ACs showed a reduced fatigue response, as expected, considering that the increase in stiffness due to the aging made mixtures more prone to fracture. However, fatigue curves representative of two ACs also in aged condition can be considered comparable (despite the experimental data being more scattered), even though the MB50/70 + A10 appears more sensitive to the strain level.

In general, the presence of bio-binder did not affect negatively the fatigue response of the studied AC. Moreover, considering the wet conditions, the bio-based AC guaranteed even better performances, confirming the AC’s low water sensitivity due to bio-binder.

Overall, test results indicate that the bio-based mixture is expected to guarantee comparable performances with the reference one, having the same penetration grade (i.e., consistency). This finding is consistent with other research studies, according to which fatigue life of AC containing bio-binders can even be improved [15,17]. Moreover, the results obtained in this study on AC mixes are in agreement with those found in previous studies focused exclusively on the binder phase [9,10] that showed comparable fatigue life for both the bio-binder and the reference bitumen.

### 4.5. Semi-Circular Bending Test

**Figure 6:**
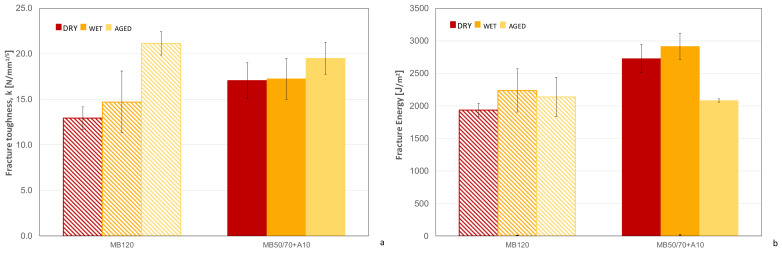
Fracture toughness (**a**) and fracture energy (**b**) results at 10 °C.

The fracture propagation behavior results of bio-based and reference AC are summarized in terms of fracture toughness (Figure 6a) and fracture energy (Figure 6b) determined at 10 °C for each testing condition (i.e., dry, wet and aged). As far as the fracture toughness is concerned, the bio-based AC showed a higher resistance to fracture (higher values of k) as compared to the reference AC in both dry and wet conditions, probably due to the slightly higher stiffness, except in the case of aged condition, where the reference AC showed a significant increase in k value. The analysis of results also shows that the MB50/70 + A10 was less dependent on the testing conditions, especially showing less sensitivity to aging effects as compared to MB120. Additionally, in terms of fracture energy G, results showed higher values for the bio-based AC as compared to the reference one. In particular, dry and wet conditions showed a better behavior compared to the reference AC, even though the improvement was not confirmed under aging conditions, for which both materials exhibited comparable values of both k and G.

### 4.6. Triaxial Cyclic Compression Test

**Figure 7:**
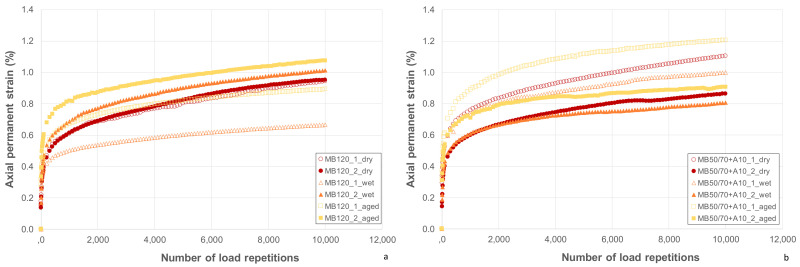
Axial permanent strain vs. number of load repetitions of aggregate concrete (AC): (**a**) MB120 and (**b**) MB50/70 + A10.

**Figure 8:**
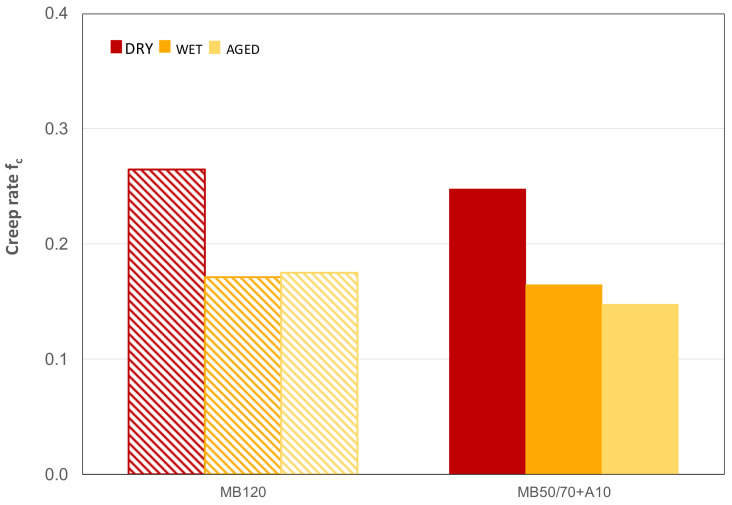
Creep rate results at 60 °C.

The creep curves used to calculate the creep rate f_c_ are shown in Figure 7a (MB120) and Figure 7b (MB50/70 + A10). Average values of permanent deformation resistance of the studied mixtures in terms of creep rate f_c_ are summarized in Figure 8. Based on the results of the creep rate, the lower f_c_, the higher the permanent deformation resistance.

Results highlight comparable performances of the two ACs regardless of testing conditions (i.e., dry, wet and aged), further confirming a similar mechanical behavior despite the use of the bio-binder and validating the hypothesis according to which the binder consistency is mainly responsible for mechanical behavior.

As far as testing conditions effects were concerned, it can be seen that wet and aged conditions led to a reduction of f_c_ of about 35% for MB120 for both conditions and of 34% and 40% for wet and aged conditions, respectively, for MB50/70 + A10. This highlights the lower tendency to accumulate permanent deformation (higher permanent deformation resistance) due to the hardening that both materials experienced in aged and wet conditions. It should also be noted that water conditioning caused a hardening effect comparable to the aging, and that the bio-based asphalt mixture showed a slightly higher aging susceptibility, confirming what emerged about stiffness characterization.

## 5. Conclusions

The current research study was aimed at evaluating the effect of a bio-binder on the performance behavior of a properly designed asphalt concrete (AC). To this end, two ACs for wearing courses having the same composition (i.e., gradation and binder content) were produced by using a bio-binder and a conventional bitumen having the same consistency, respectively. An extensive laboratory characterization was performed to evaluate and compare the mechanical behavior of the ACs. In addition, aging and water sensitivity of ACs were evaluated.

Based upon the experimental results, the following conclusions can be drawn:

- *ITS*, *ITSM* and TCC results show that the mechanical response of bio-binder-based AC is comparable to the reference AC in all testing conditions considered; however, the bio-based AC seemed to show a slightly higher sensitivity to the hardening due to aging effects.
- The presence of the investigated bio-binder did not affect the temperature dependence of the asphalt mixture, showing similar sensitivity in all testing conditions (i.e., dry, wet, and aged), highlighting the principal role of the binder consistency.
- Although *ITSM* results highlighted that bio-based AC seems to be more prone to age-hardening (stiffening), fatigue and SCB tests showed that the fatigue response of bio-based AC was still comparable to that of the reference mixture and resistance to fracture was comparable or even improved at low temperature.
- Similarly to the reference mixture, no water susceptibility can be attributed to the bio-based AC, however noting that moisture sensitivity observed depended on the volumetric characteristics of specimens.

Overall, no substantial differences were found in the mechanical performances of the two investigated ACs regardless of selected testing conditions. In conclusion, it can be stated that the use of the selected bio-oil-based binder to produce AC did not show any reduced mechanical properties and it was perfectly comparable to the conventional AC produced with a normal bitumen. Therefore, the current findings encourage the use of such bio-binders as a valid alternative to traditional asphalt mixtures; this also matches sustainability needs in road construction.

As for future work, in order to thoroughly assess the applicability of such asphalt mixtures made with bio-binder, further investigations could be done on asphalt cores taken from a field trial.

## Figures and Tables

**Table 1:** Characteristics of the binders employed in the asphalt mixtures.

Binder	Penetration (0.1 mm)	Softening Point (°C)	Viscosity 160 °C (mPa·s)
B.50/70 + A10	120	45.3	112.8
B.120	120	45.0	105.5

**Table 2:** Summary of testing program.

Mixture	*ITS*			*ITSM*			ITFT			SCB			TCC		
	Dry	Wet	Aged	Dry	Wet	Aged	Dry	Wet	Aged	Dry	Wet	Aged	Dry	Wet	Aged
MB50/70 + A10	4	4	4	9	9	9	6	6	6	4	4	4	2	2	2
MB120	4	4	4	9	9	9	6	6	6	4	4	4	2	2	2

**Table 3:** Compaction results in terms of number of rotations.

Material	Number of Gyrations		ANOVA	
	Avg. Value (-)	Std. Dev. (-)	*p*-Value	Significant
MB120	37.8	9.6	0.167	NO
MB50/70 + A10	35.1	9.4	-	-

**Table 4:** Indirect tensile stiffness modulus (*ITSM*) variation results in wet and aged conditions for both ACs.

	**MB50/70 + A10**		**MB120**	
	$\frac{ITSM_{wet}}{ITSM_{dry}}$	$\frac{ITSM_{aged}}{ITSM_{dry}}$	$\frac{ITSM_{wet}}{ITSM_{dry}}$	$\frac{ITSM_{aged}}{ITSM_{dry}}$
10 °C	1.25	1.62	1.04	1.31
20 °C	1.27	1.83	1.12	1.46
40 °C	1.27	2.29	1.15	1.91

**Table 5:** Equations of the fatigue laws.

Mixture	Dry	Wet	Aged
MB50/70 + A10	ε = 2393.4 Nf ^−0.196^	ε = 2279.4 Nf ^−0.182^	ε = 1945.7 Nf ^−0.192^
MB120	ε = 2023 Nf ^−0.182^	ε = 1893 Nf ^−0.171^	ε = 1212.8 Nf ^−0.142^

## Data Availability

The data presented in this study are available on request from the corresponding author.
